# Supplementary figures and images for: Diatom heterotrophy on brown algal polysaccharides emerged through horizontal gene transfer, gene duplication, and neofunctionalization
Source: PLoS Biol. 2025 Apr 1;23(4):e3003038. doi: 10.1371/journal.pbio.3003038 (PMC11960938; doi:10.1371/journal.pbio.3003038)

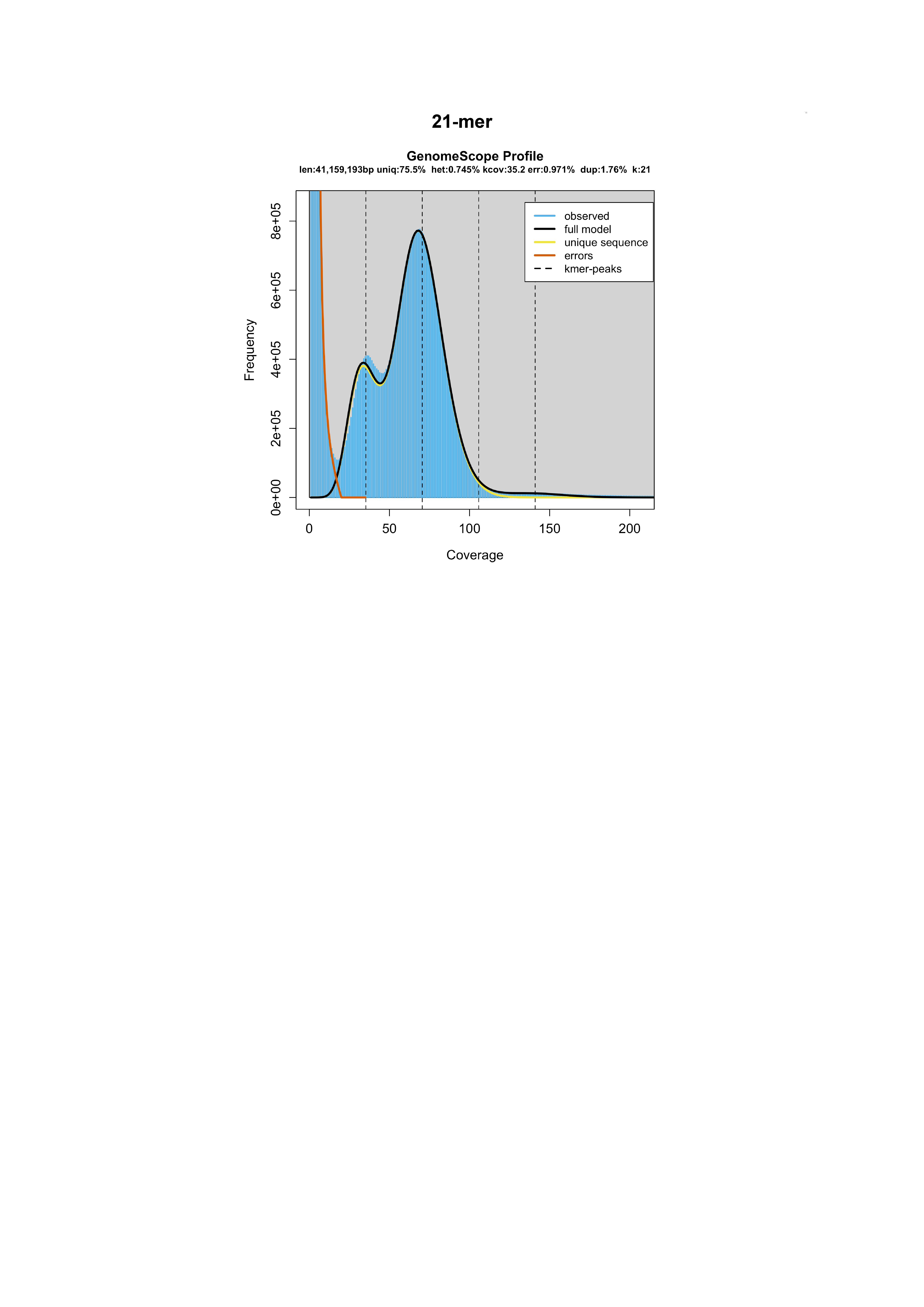

Supplement: S1 Fig — 21-mers (k-mers) were counted using the tool Jellyfish and analyzed with GenomeScope. The presence of two major peaks observed in the GenomeScope profile suggests that N. sing1 is diploid. The data underlying this figure can be found in S1 Data. (TIF) [file pbio.3003038.s001.tif]

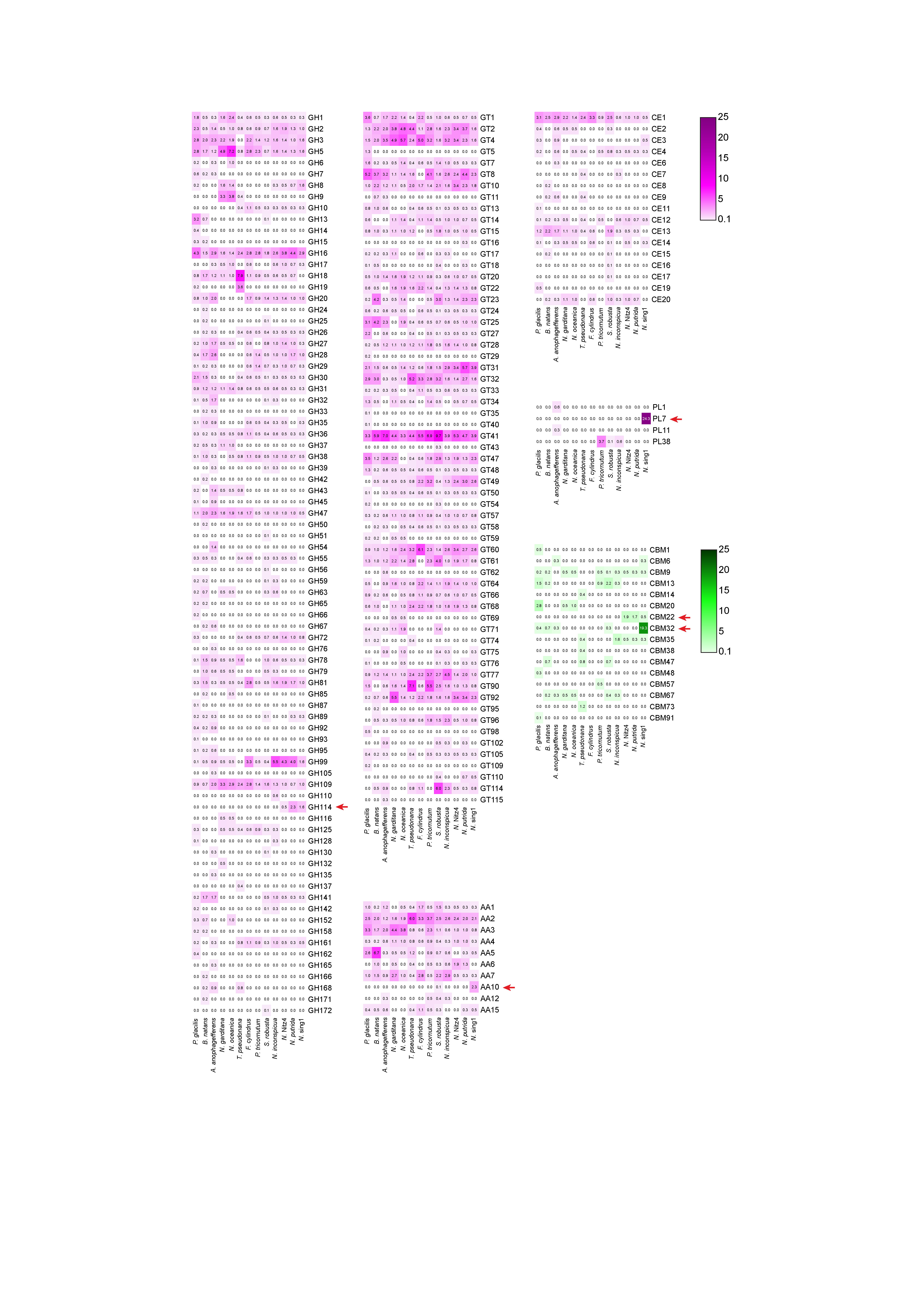

Supplement: S2 Fig — The heatmap displays the proportion of genes annotated with CAZyme family domains (CAZyme family genes, magenta scale) or carbohydrate-binding domains (CBM-containing genes, green scale) as a percentage of the total CAZyme genes annotated in each species. Red arrows denote families that may have undergone expansion in the apochlorotic or N. sing1 lineage. The data underlying this figure can be found in S2 Data. (TIF) [file pbio.3003038.s002.tif]

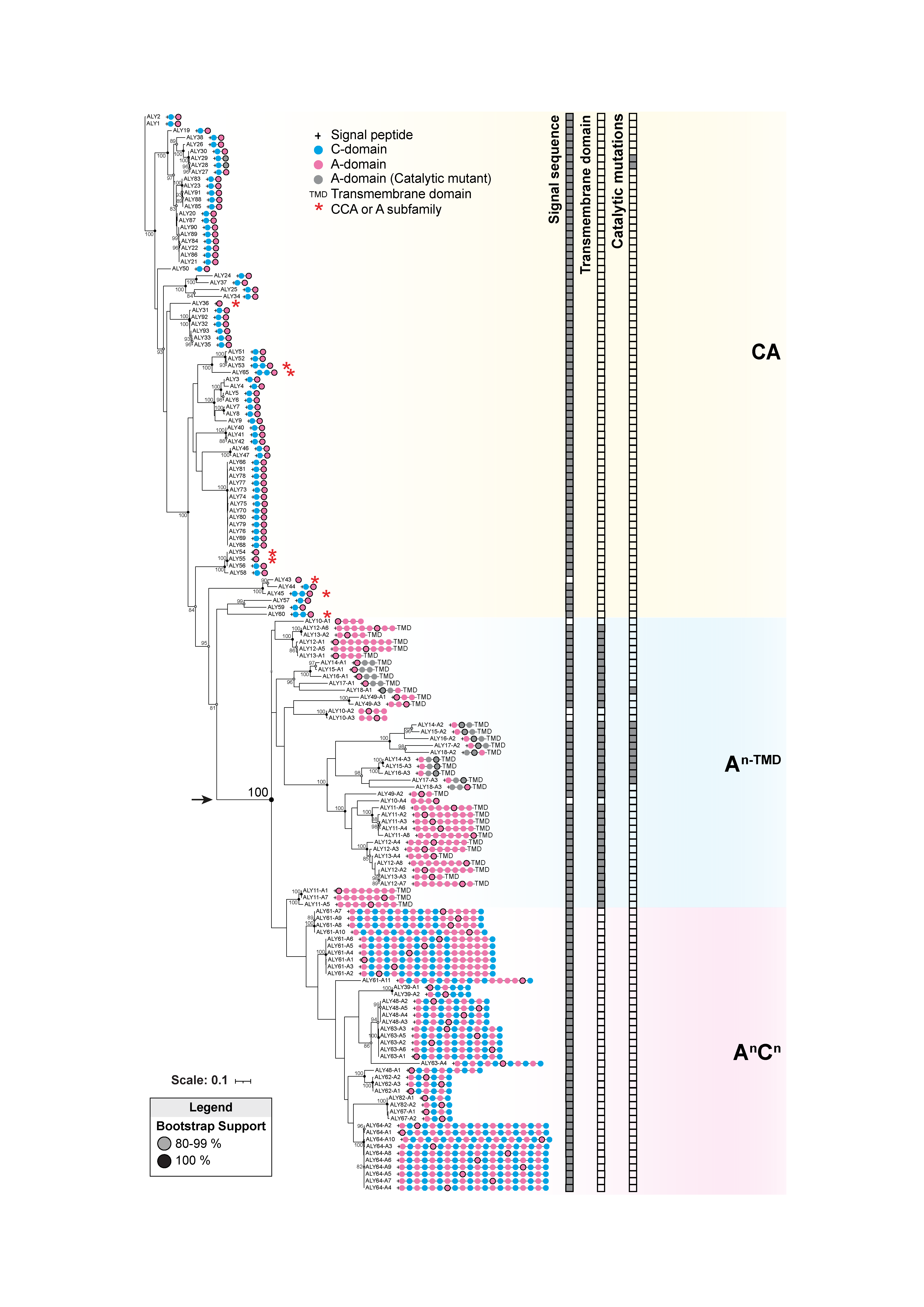

Supplement: S3 Fig — The phylogenetic tree (bootstrap replicates = 1000) was constructed using the nucleotide sequences of all N. sing1 ALY A-domains. Bootstrap support for nodes is summarized in the legend. Tree scale = 0.1. Domain organization of the ALY gene is illustrated next to each leaf. The domain corresponding to the leaf is identified by a black border. Domains belonging to the CCA or A subfamily ALYs are marked with a red asterisk. The presence/absence of signal peptide and transmembrane domain (TMD) in the ALY gene each A-domain belongs to, along with whether the domain contains mutations at key catalytic sites are summarized on the right. Clades highlighted in yellow, blue and red fall under the CA, An-TMD and AnCn families, respectively. The node giving rise to the derived ALY families (An-TMD and AnCn) is marked with a black arrow. The data underlying this figure can be found in S5 Data. (TIF) [file pbio.3003038.s003.tif]

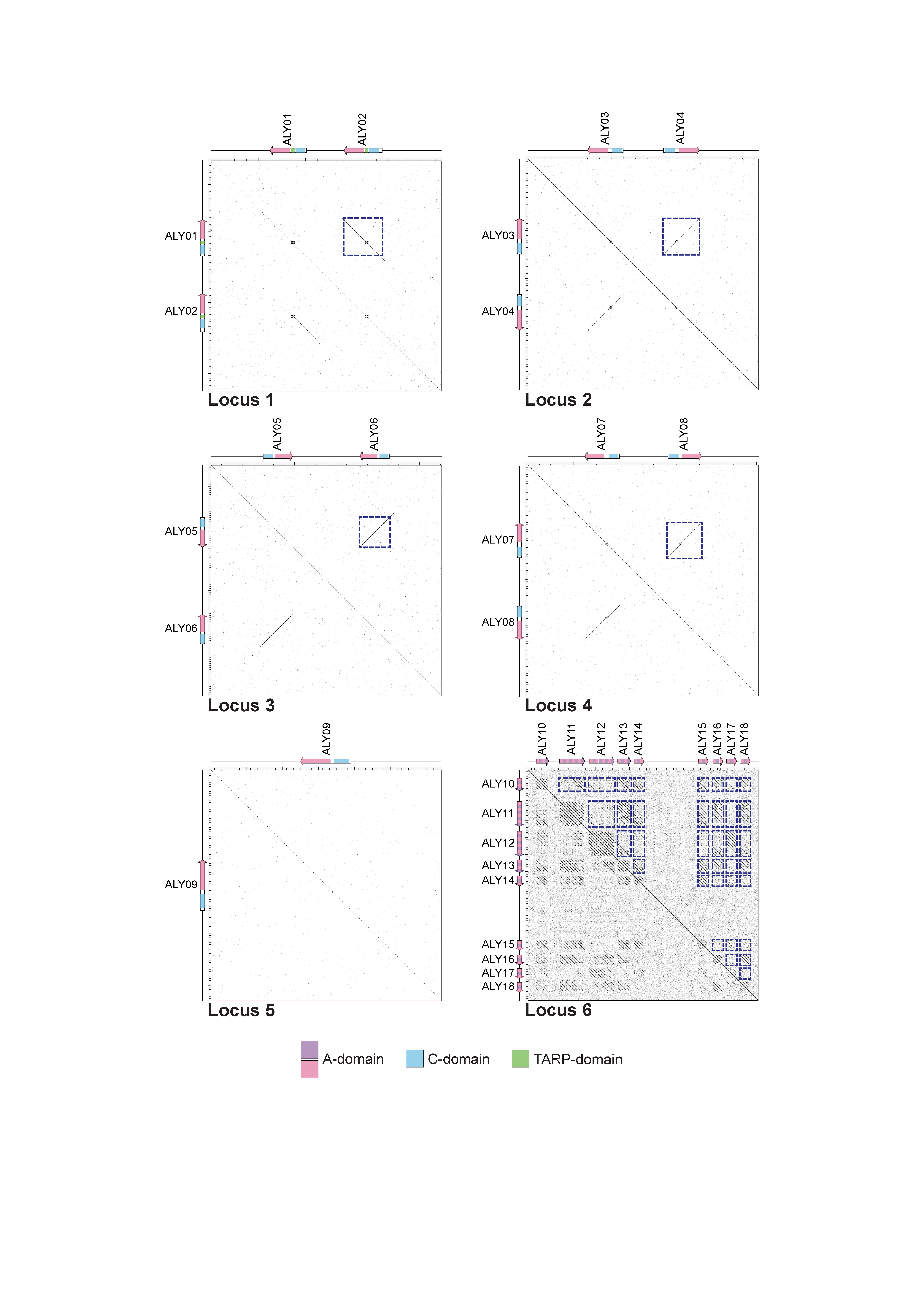

Supplement: S4 Fig — Self-alignment of ALY loci 1–6 nucleotide sequence (± 2.5 kb) reveals patterns of DNA homology within each locus. The gene structure of the ALY locus is shown along both axes. Protein domains are labeled according to the legend. Dashed dark blue and red boxes identify homology between genic and intergenic regions of the locus, respectively. (TIF) [file pbio.3003038.s004.tif]

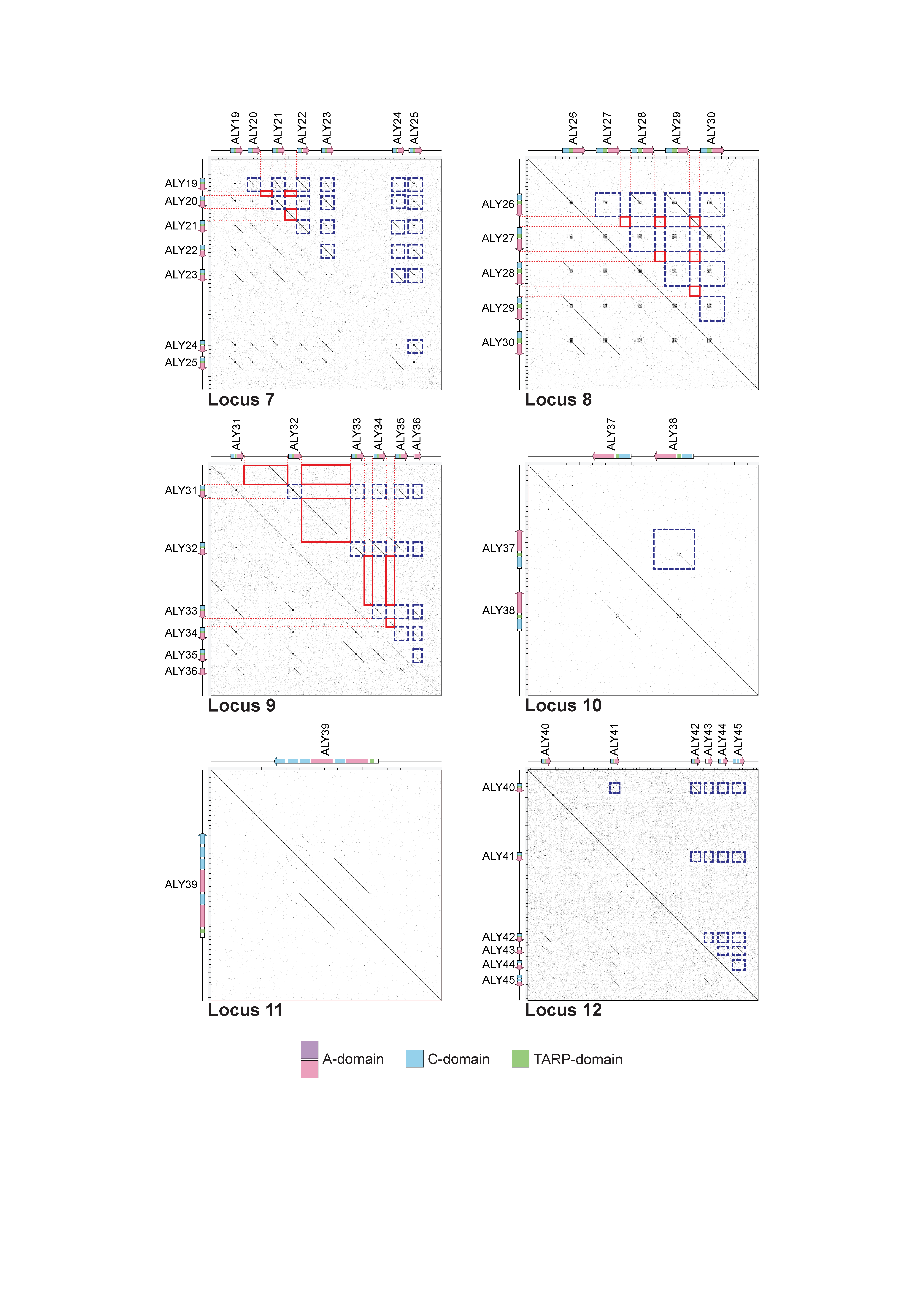

Supplement: S5 Fig — Self-alignment of ALY loci 7–12 nucleotide sequence ( ± 2.5 kb) reveals patterns of DNA homology within each locus. The gene structure of the ALY locus is shown along both axes. Protein domains are labeled according to the legend. Dashed dark blue and red boxes identify homology between genic and intergenic regions of the locus, respectively. (TIF) [file pbio.3003038.s005.tif]

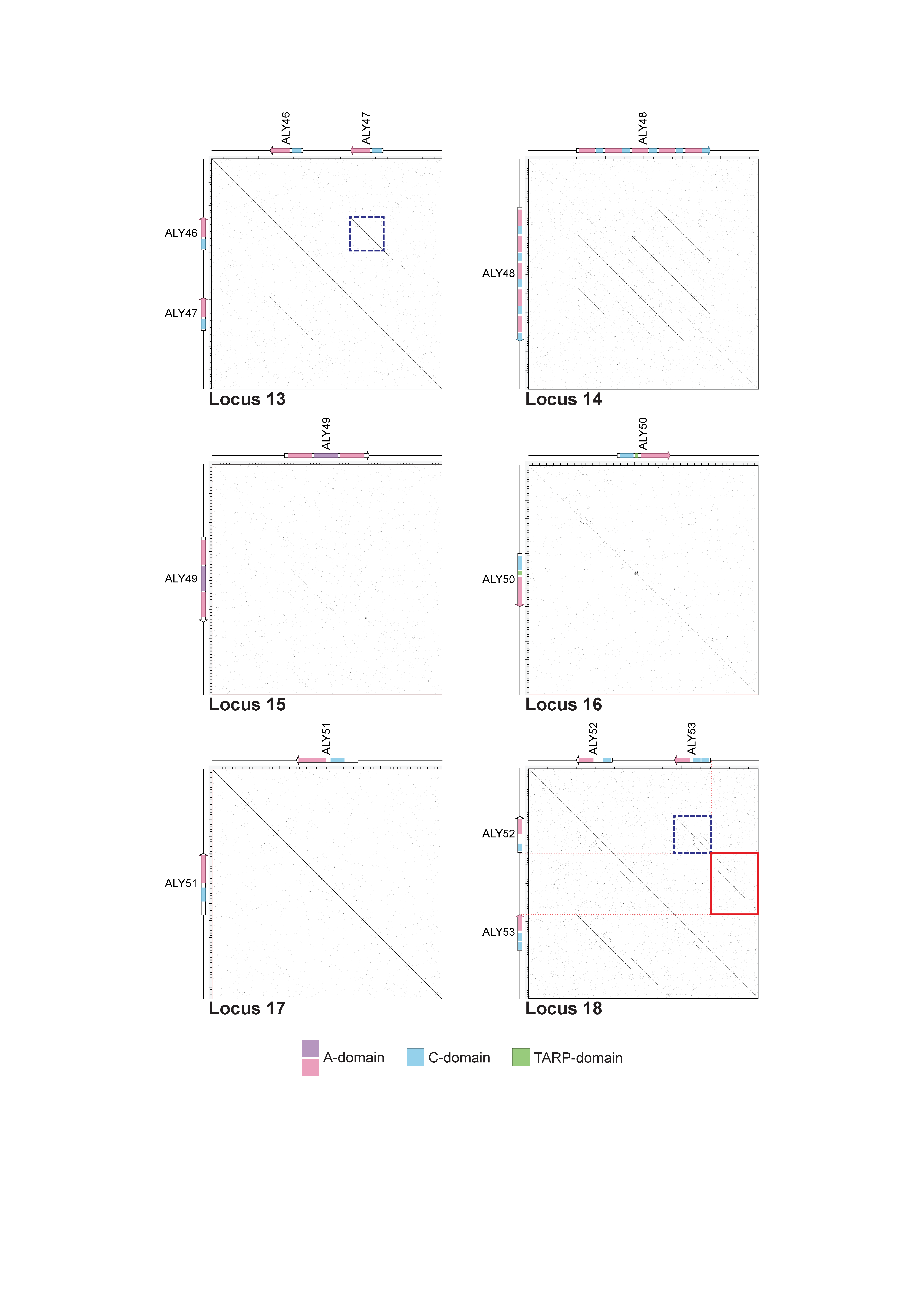

Supplement: S6 Fig — Self-alignment of ALY loci 13–18 nucleotide sequence ( ± 2.5 kb) reveals patterns of DNA homology within each locus. The gene structure of the ALY locus is shown along both axes. Protein domains are labeled according to the legend. Dashed dark blue and red boxes identify homology between genic and intergenic regions of the locus, respectively. (TIF) [file pbio.3003038.s006.tif]

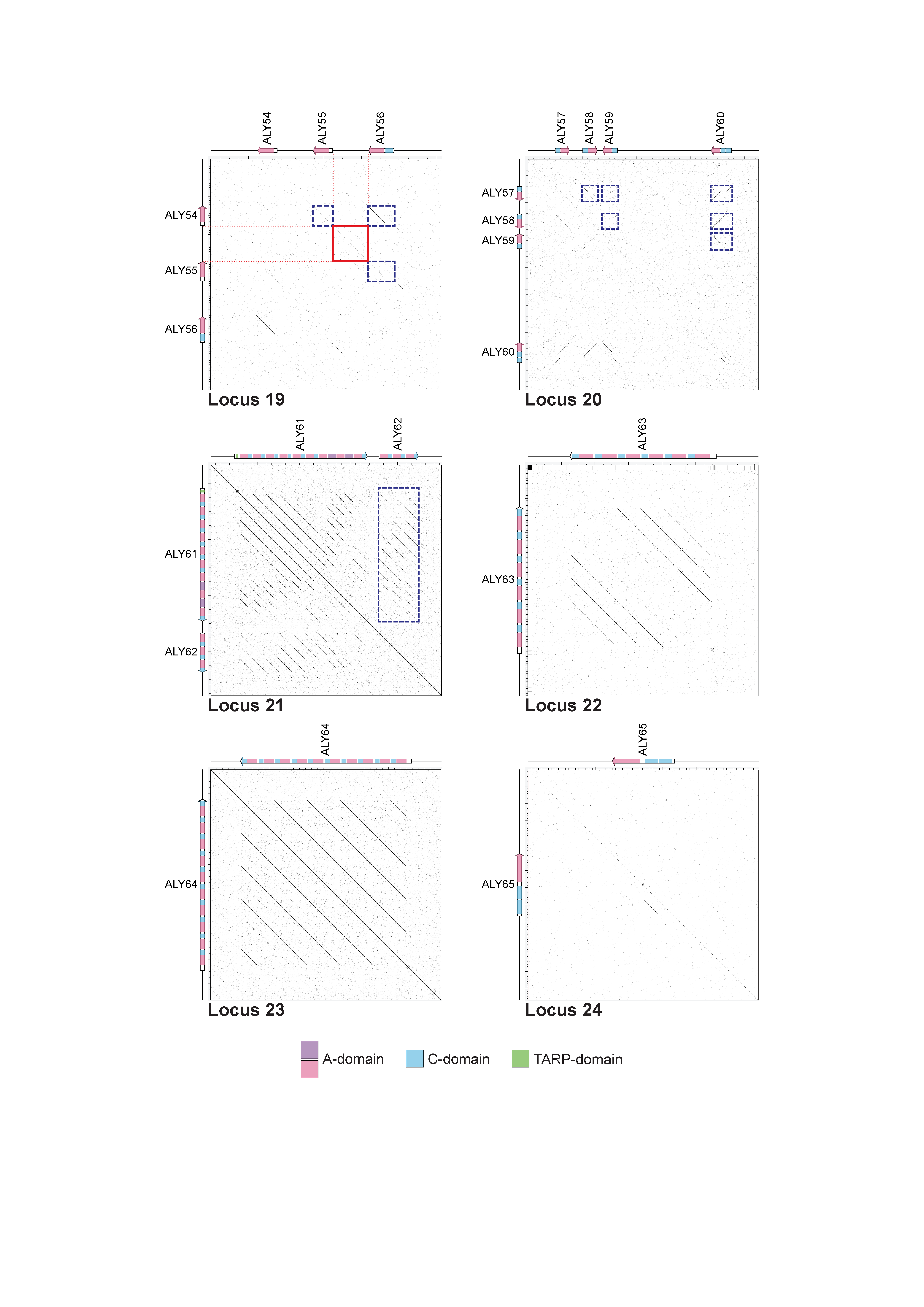

Supplement: S7 Fig — Self-alignment of ALY loci 19–24 nucleotide sequence ( ± 2.5 kb) reveals patterns of DNA homology within each locus. The gene structure of the ALY locus is shown along both axes. Protein domains are labeled according to the legend. Dashed dark blue and red boxes identify homology between genic and intergenic regions of the locus, respectively. (TIF) [file pbio.3003038.s007.tif]

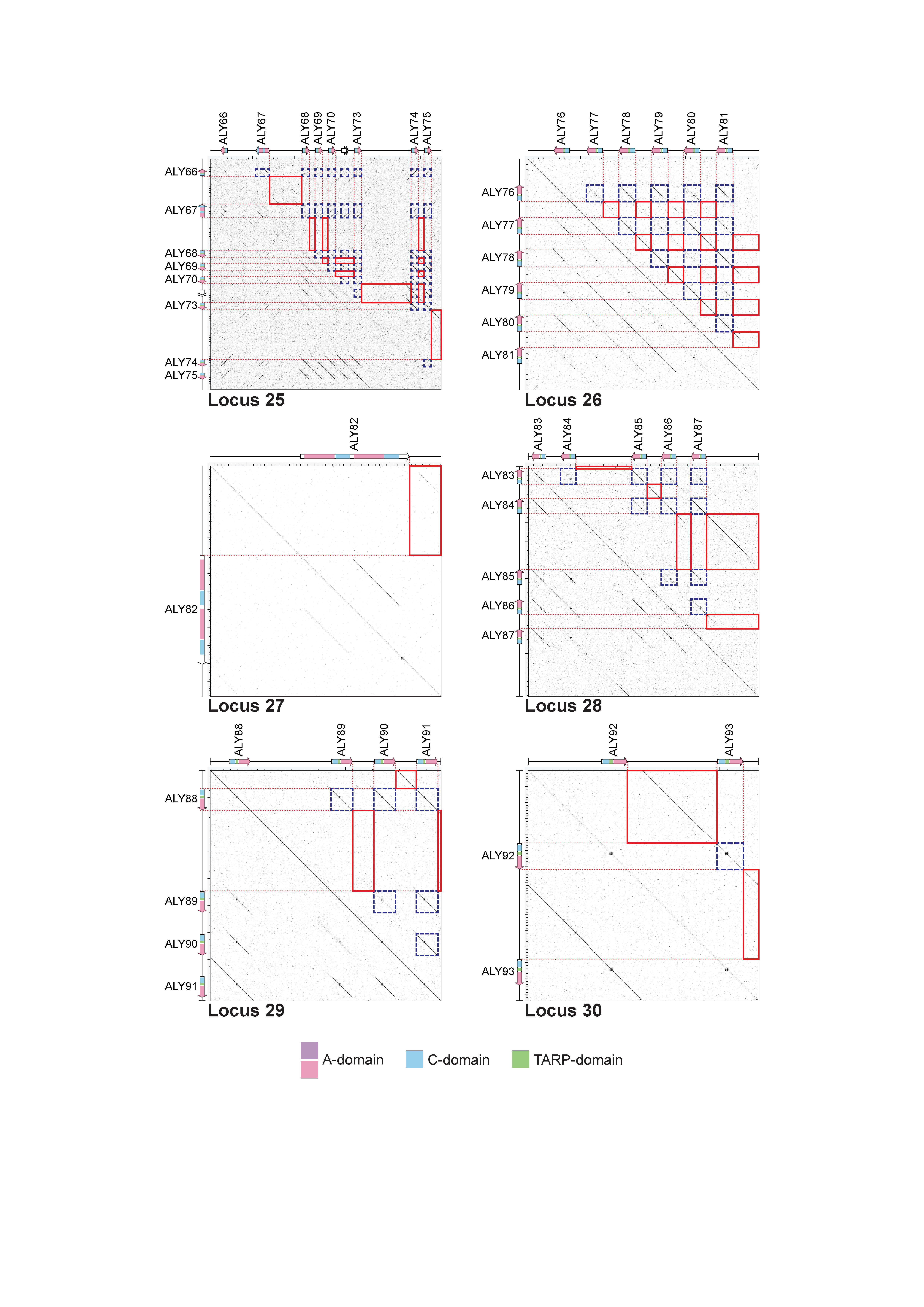

Supplement: S8 Fig — Self-alignment of ALY loci 25–30 nucleotide sequence ( ± 2.5 kb) reveals patterns of DNA homology within each locus. The gene structure of the ALY locus is shown along both axes. Protein domains are labeled according to the legend. Dashed dark blue and red boxes identify homology between genic and intergenic regions of the locus, respectively. (TIF) [file pbio.3003038.s008.tif]

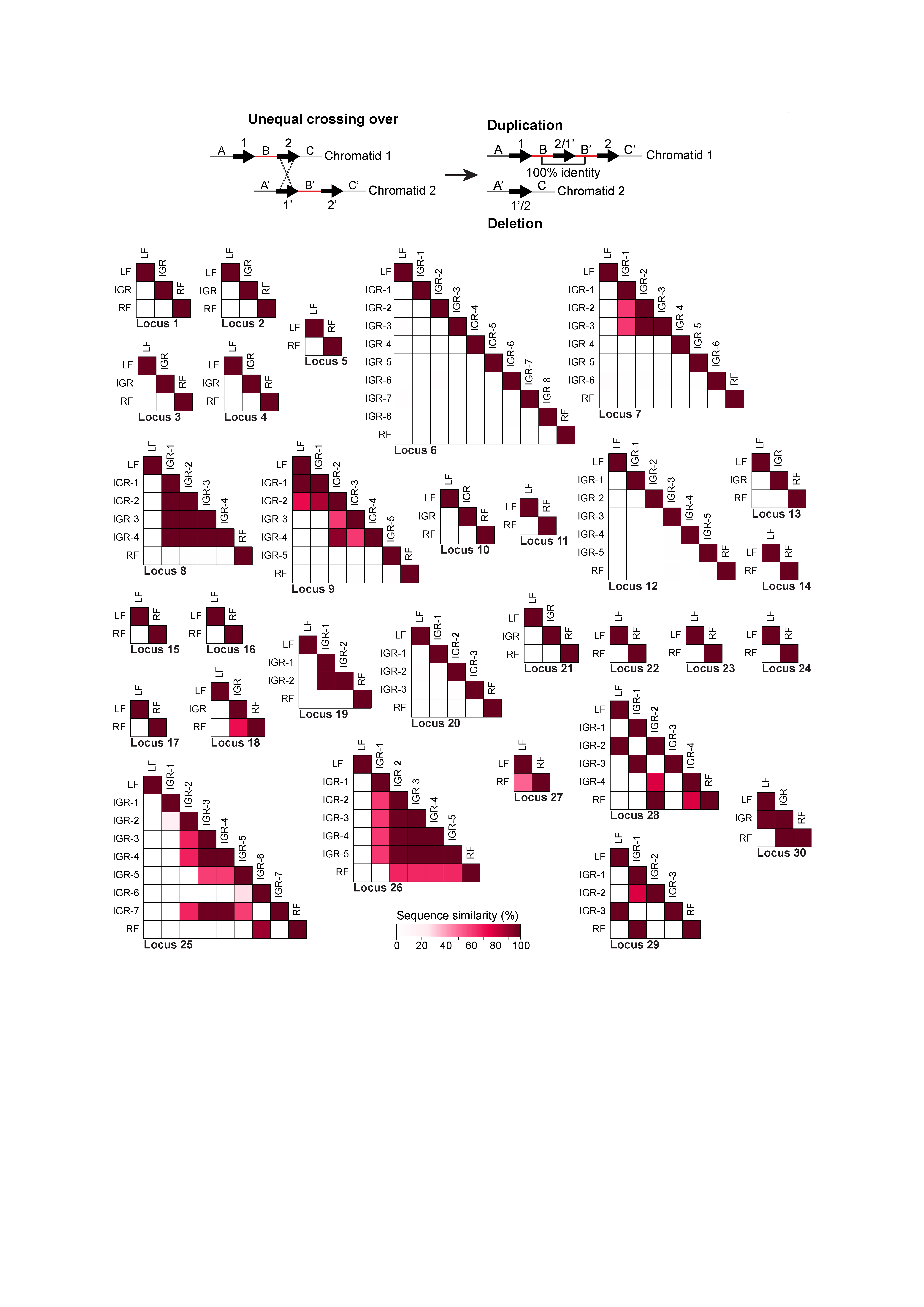

Supplement: S9 Fig — (Top) The cartoon illustrates how unequal crossing-over at a locus with two tandem genes can lead to both duplication of the gene and intergenic region. (Bottom) Self-comparisons of intergenic sequences within all N. sing1 ALY loci (loci 1–30). The percent sequence identity for each pairwise comparison is shown as a matrix plot. IGR, intergenic region; LF, left flank; RF, right flank. The data underlying this figure can be found in S6 Data. (TIF) [file pbio.3003038.s009.tif]

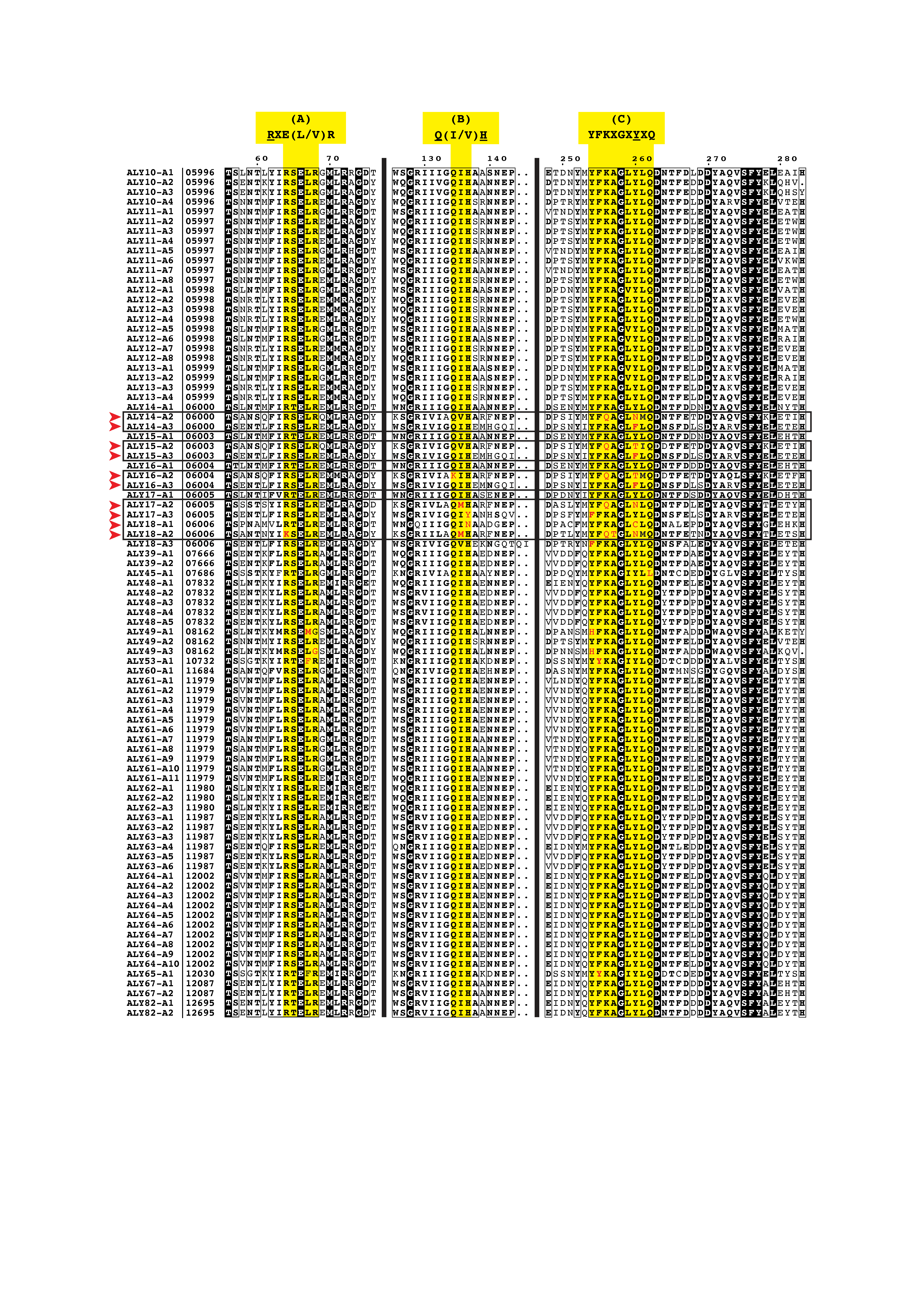

Supplement: S10 Fig — Protein sequence alignment of An-TMD and AnCn family ALYs (A-domain catalytic regions; A–C) in N. sing1. Conserved catalytic residues are highlighted in yellow (A: RXE(L/V)R; B: Q(I/V)H; and C: YFKXGXYXQ). The red arrowheads denote A-domains with substitutions at key catalytic residues in at least one of these three catalytic regions. (TIF) [file pbio.3003038.s010.tif]

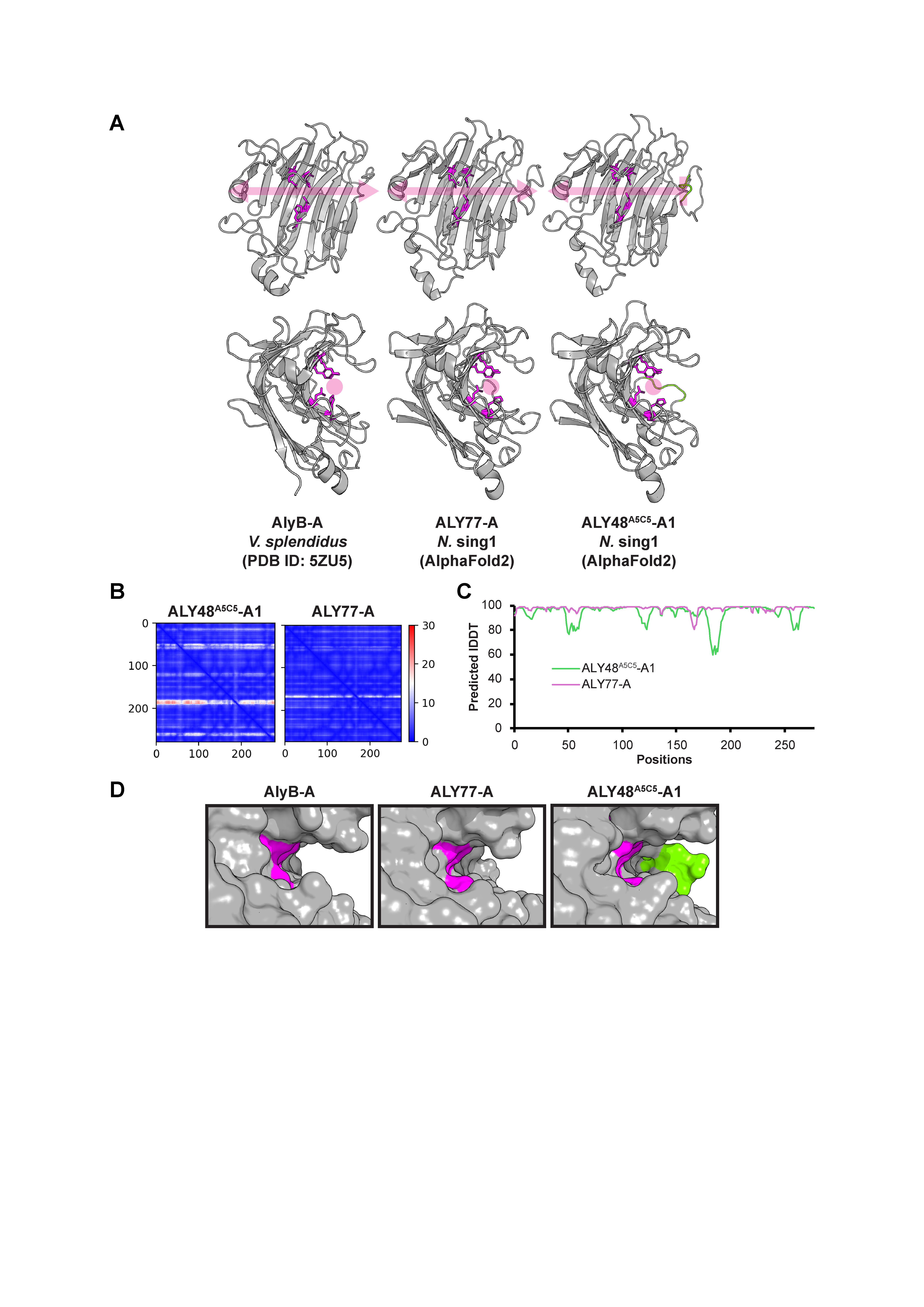

Supplement: S11 Fig — (A) (Left to right) Cartoon representations of the protein crystal structure of AlyB alginate lyase domain (V. splendidus; PDB 5ZU5) and AlphaFold2 predictions of ALY77 and ALY48A5C5 A-domains (N. sing1; ALY77-A and ALY48A5C5-A1). Top and bottom rows show two different views along the catalytic cleft. Putative catalytic residues (R/Q/H/Y) are colored in magenta, while the 15-bp insertion predicted to contribute to exolytic activity is colored in green. Magenta arrows and circles indicate the likely binding site of alginate molecules along the catalytic cleft. The data underlying this figure can be found at https://zenodo.org/records/14793551. (B) Predicted aligned error (PAE) plots of the two AlphaFold2-predicted structures in (A) show the confidence in the relative position of two residues within the predicted structure. The data underlying this figure can be found in S8 Data. (C) Predicted local distance difference test (pLDDT) scores for each residue of the two AlphaFold2-predicted structures in (A), which signifies the confidence scores of the prediction. The data underlying this figure can be found in S8 Data. (D) Magnified view of the alginate lyase catalytic groove (displayed as surface representations). Note that the loop insertion in ALY48A5C5-A1 appears to block the groove. (TIF) [file pbio.3003038.s011.tif]

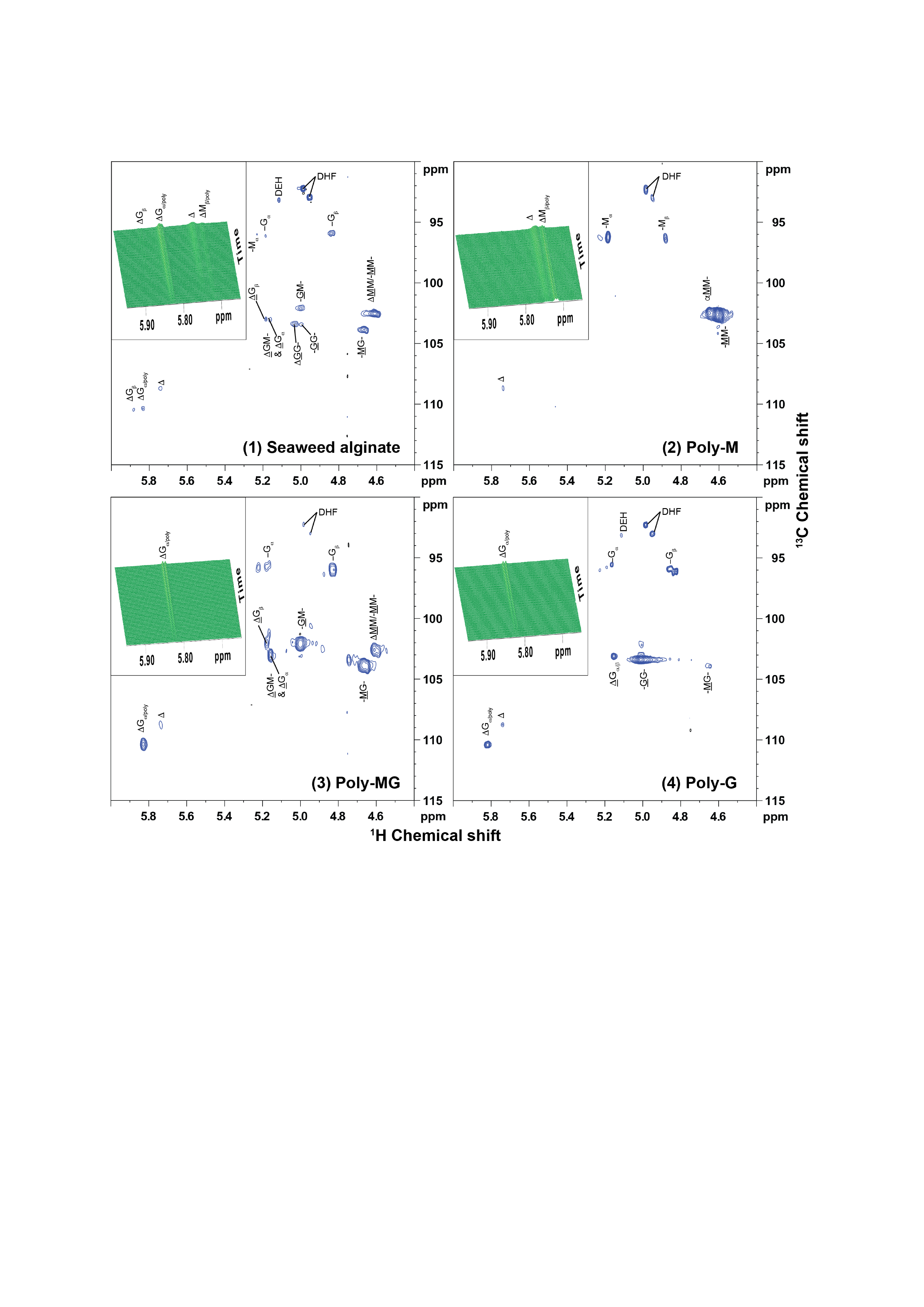

Supplement: S12 Fig — Anomeric and ∆ H/C-4 regions of 1H-13C HSQC of (1) Seaweed alginate (FG 0.46; DP ~30) (top left panel), (2) Poly-M (DP ~30) (top right panel), (3) Poly-MG (DP ~26) (bottom left panel), and (4) Poly-G (DP ~26) (bottom right panel) treated with ALY48A5C5-A1 after 5 h 20 min (in 20 mM HEPES (pH 7.5), 25 mM NaCl, 2 mM CaCl2 in D2O (D, 99.9%) recorded on a 600 or 800 MHz instrument at 25 °C. The inlay panels show 1H time-resolved spectrum of the ∆H/C-4 region over the reaction period. M: Mannuronate; G: Guluronate; ∆: ∆H/C-4 of 4,5-unsaturated 4-deoxy-l-erythro-hex-4-enepyranosyluronate; ∆: ∆H/C-1 (anomeric signal) of 4,5-unsaturated 4-deoxy-l-erythro-hex-4-enepyranosyluronate; DEH: 4-deoxy-l-erythro-5-hexulosuronate hydrate; DHF: two epimers 4-deoxy-d-manno-(5S)-hexulofuranosidonate hydrate and 4-deoxy-d-manno-(5R)-hexulofuranosidonate hydrate; α/β: M/G at reducing ends of alginate residue; poly: M/G in a polymer; GM-/MG-: Alternating GM/MG polymer. Underlined labels indicate the residue with the anomeric H/C-1 giving rise to the signal, and -xx- indicates signals within a polysaccharide chain. The data underlying this figure can be found at https://zenodo.org/records/14410447. (TIF) [file pbio.3003038.s012.tif]

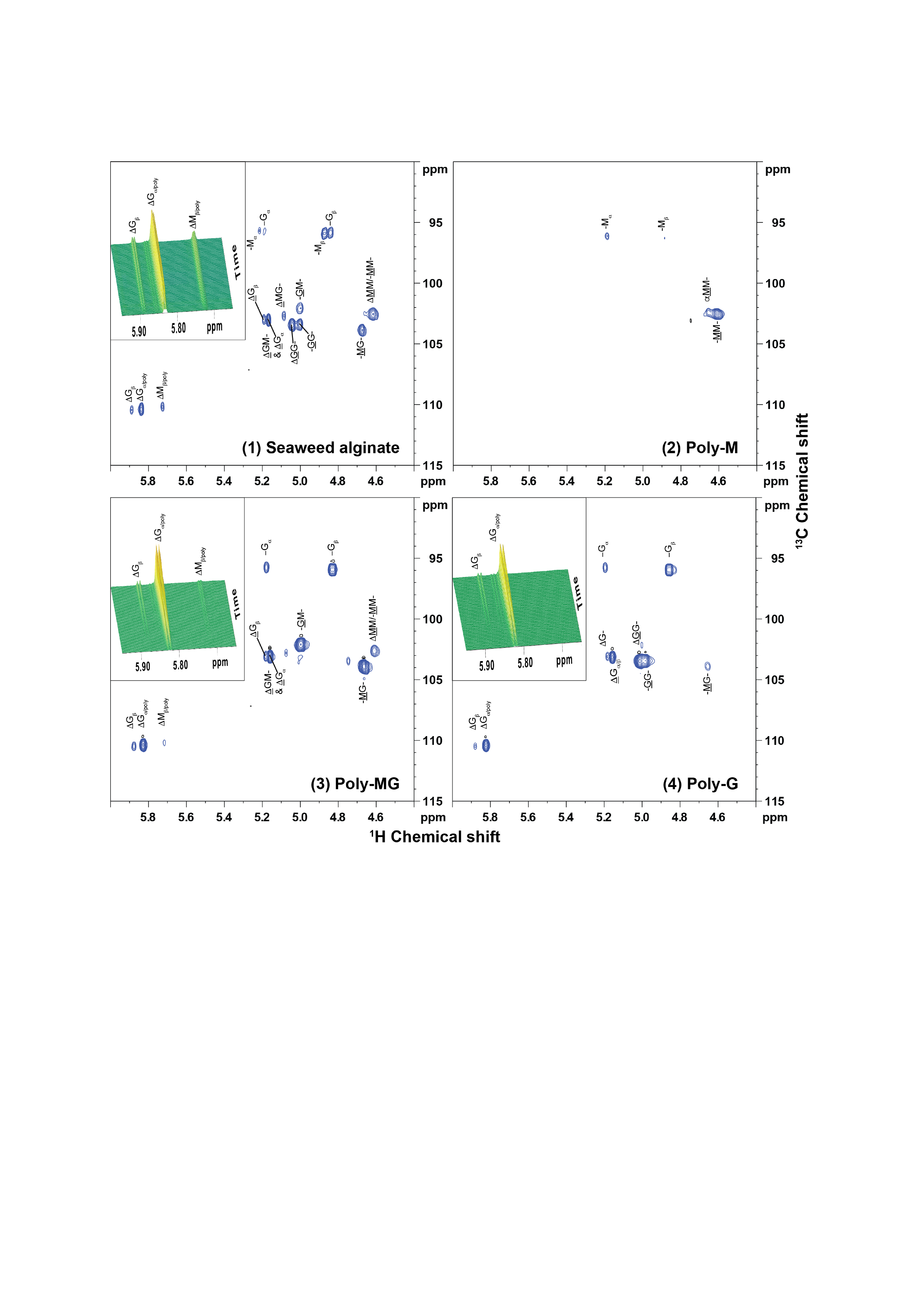

Supplement: S13 Fig — Anomeric and ∆H/C-4 regions of 1H-13C HSQC of (1) Seaweed alginate (FG 0.46; DP ~30) (top left panel), (2) Poly-M (DP ~30) (top right panel), (3) Poly-MG (DP ~26) (bottom left panel), and (4) Poly-G (DP ~26) (bottom right panel) treated with ALY7-A after 5 h 20 min (in 20 mM HEPES (pH 7.5), 25 mM NaCl, 2 mM CaCl2 in D2O (D, 99.9%) recorded on a 600 or 800 MHz instrument at 25 °C. The inlay panels show 1H time-resolved spectrum of the ∆H/C-4 region over the reaction period. The inlay panel for (2) Poly-M is omitted due to the absence of activity. M: Mannuronate; G: Guluronate; ∆: ∆H/C-4 of 4,5-unsaturated 4-deoxy-l-erythro-hex-4-enepyranosyluronate; ∆: ∆H/C-1 (anomeric signal) of 4,5-unsaturated 4-deoxy-l-erythro-hex-4-enepyranosyluronate; DEH: 4-deoxy-l-erythro-5-hexulosuronate hydrate; DHF: two epimers 4-deoxy-d-manno-(5S)-hexulofuranosidonate hydrate and 4-deoxy-d-manno-(5R)-hexulofuranosidonate hydrate; α/β: M/G at reducing ends of alginate residue; poly: M/G in a polymer; GM-/MG-: Alternating GM/MG polymer. Underlined labels indicate the residue with the anomeric H/C-1 giving rise to the signal, and -xx- indicates signals within a polysaccharide chain. The data underlying this figure can be found at https://zenodo.org/records/14410447. (TIF) [file pbio.3003038.s013.tif]

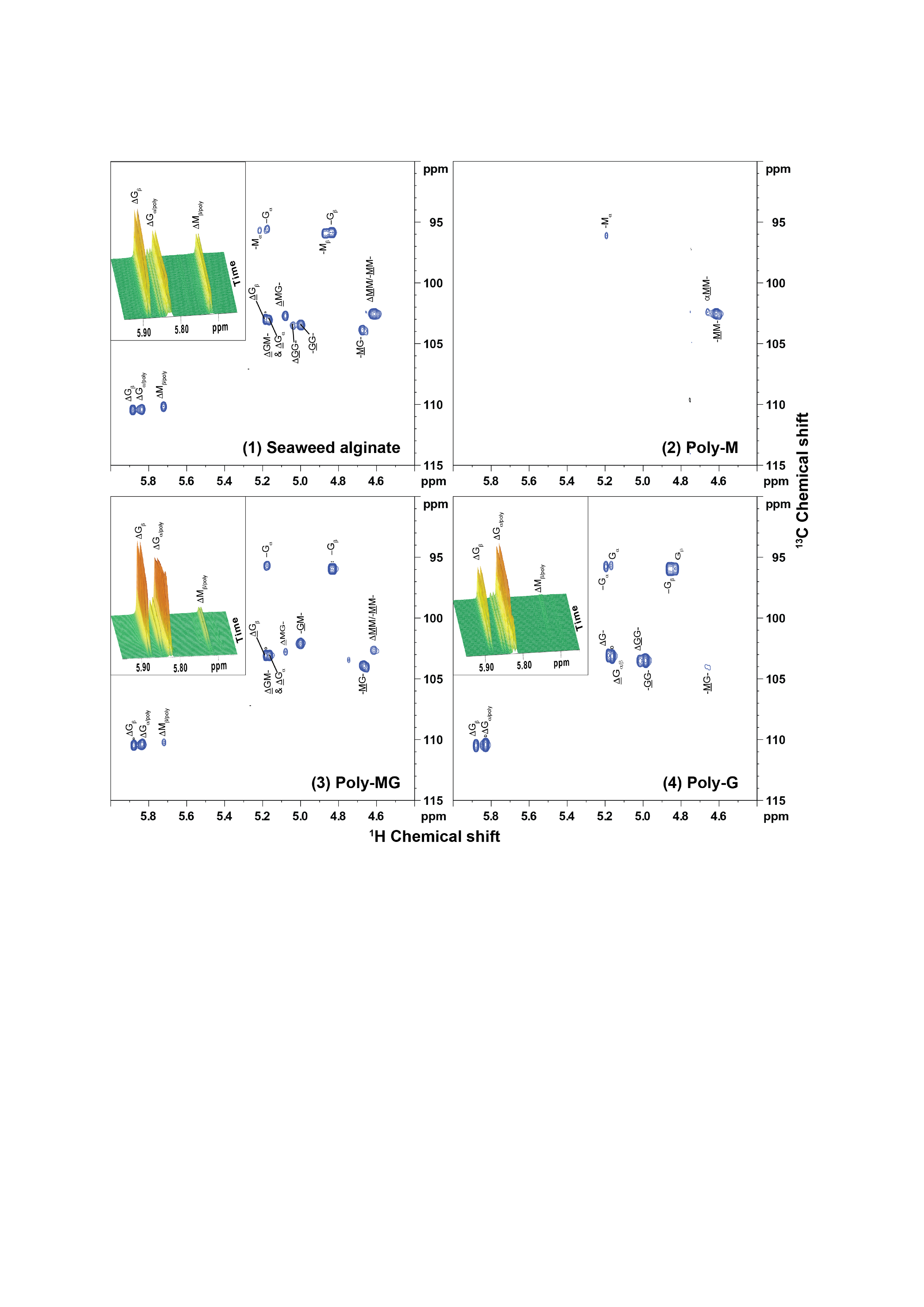

Supplement: S14 Fig — Anomeric and ∆H/C-4 regions of 1H-13C HSQC of (1) Seaweed alginate (FG 0.46; DP ~30) (top left panel), (2) Poly-M (DP ~30) (top right panel), (3) Poly-MG (DP ~26) (bottom left panel), and (4) Poly-G (DP ~26) (bottom right panel) treated with ALY58-A after 5 h 20 min (in 20 mM HEPES (pH 7.5), 25 mM NaCl, 2 mM CaCl2 in D2O (D, 99.9%) recorded on a 600 or 800 MHz instrument at 25°C. The inlay panels show 1H time-resolved spectrum of the ∆H/C-4 region over the reaction period. The inlay panel for (2) Poly-M is omitted due to the absence of activity. M: Mannuronate; G: Guluronate; ∆: ∆H/C-4 of 4,5-unsaturated 4-deoxy-l-erythro-hex-4-enepyranosyluronate; ∆: ∆H/C-1 (anomeric signal) of 4,5-unsaturated 4-deoxy-l-erythro-hex-4-enepyranosyluronate; DEH: 4-deoxy-l-erythro-5-hexulosuronate hydrate; DHF: two epimers 4-deoxy-d-manno-(5S)-hexulofuranosidonate hydrate and 4-deoxy-d-manno-(5R)-hexulofuranosidonate hydrate; α/β: M/G at reducing ends of alginate residue; poly: M/G in a polymer; GM-/MG-: Alternating GM/MG polymer. Underlined labels indicate the residue with the anomeric H/C-1 giving rise to the signal, and -xx- indicates signals within a polysaccharide chain. The data underlying this figure can be found at https://zenodo.org/records/14410447. (TIF) [file pbio.3003038.s014.tif]

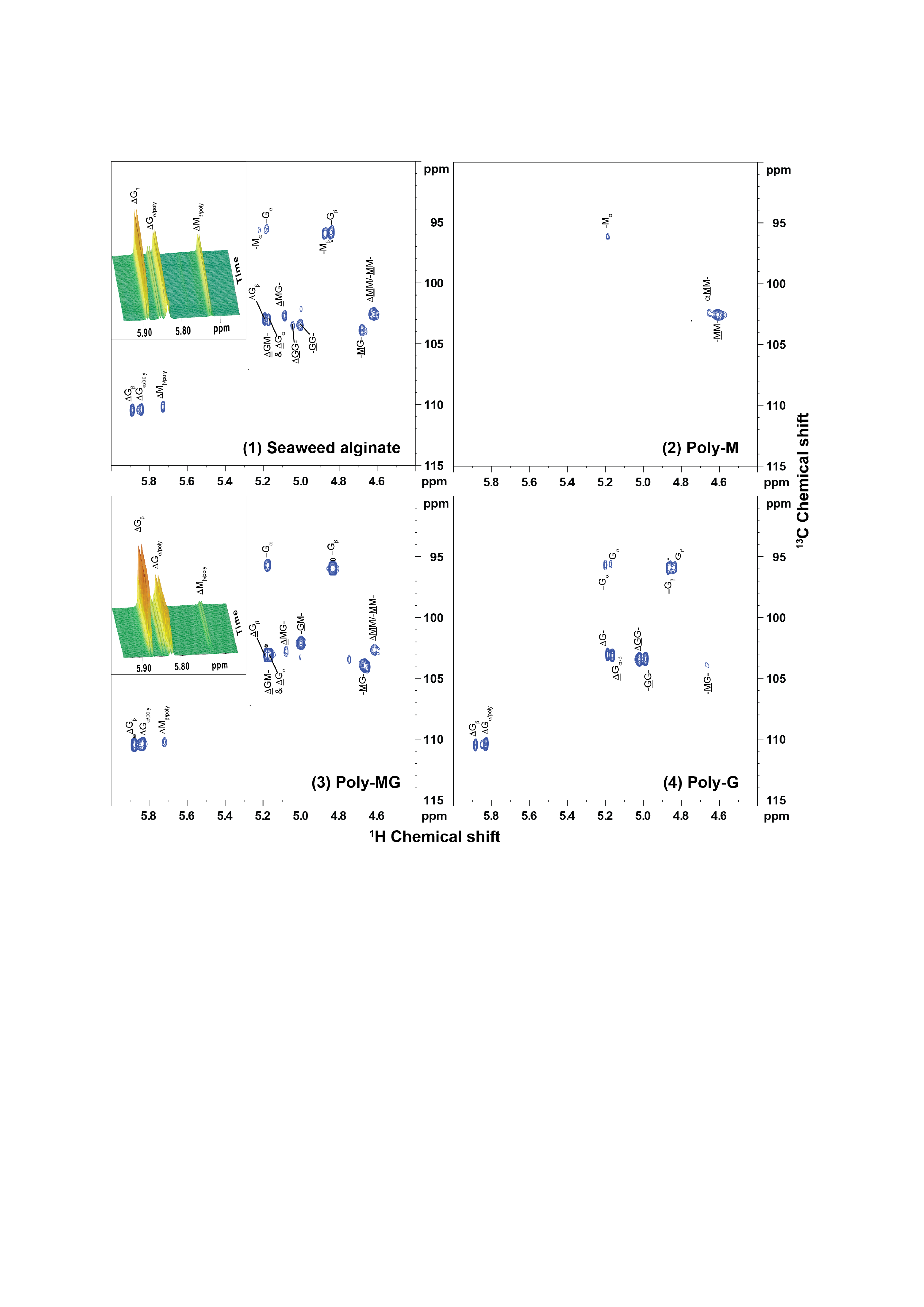

Supplement: S15 Fig — Anomeric and ∆H/C-4 regions of 1H-13C HSQC of (1) Seaweed alginate (FG 0.46; DP ~30) (top left panel), (2) Poly-M (DP ~30) (top right panel), (3) Poly-MG (DP ~26) (bottom left panel), and (4) Poly-G (DP ~26) (bottom right panel) treated with ALY77-A after 5 h 20 min (in 20 mM HEPES (pH 7.5), 25 mM NaCl, 2 mM CaCl2 in D2O (D, 99.9%) recorded on a 600 or 800 MHz instrument at 25 °C. The inlay panels show 1H time-resolved spectrum of the ∆H/C-4 region over the reaction period. The inlay panel for (2) Poly-M and (4) Poly-G is omitted due to the absence of activity and lock error during recording, respectively. M: Mannuronate; G: Guluronate; ∆: ∆H/C-4 of 4,5-unsaturated 4-deoxy-l-erythro-hex-4-enepyranosyluronate; ∆: ∆H/C-1 (anomeric signal) of 4,5-unsaturated 4-deoxy-l-erythro-hex-4-enepyranosyluronate; DEH: 4-deoxy-l-erythro-5-hexulosuronate hydrate; DHF: two epimers 4-deoxy-d-manno-(5S)-hexulofuranosidonate hydrate and 4-deoxy-d-manno-(5R)-hexulofuranosidonate hydrate; α/β: M/G at reducing ends of alginate residue; poly: M/G in a polymer; GM-/MG-: Alternating GM/MG polymer. Underlined labels indicate the residue with the anomeric H/C-1 giving rise to the signal, and -xx- indicates signals within a polysaccharide chain. The data underlying this figure can be found at https://zenodo.org/records/14410447. (TIF) [file pbio.3003038.s015.tif]

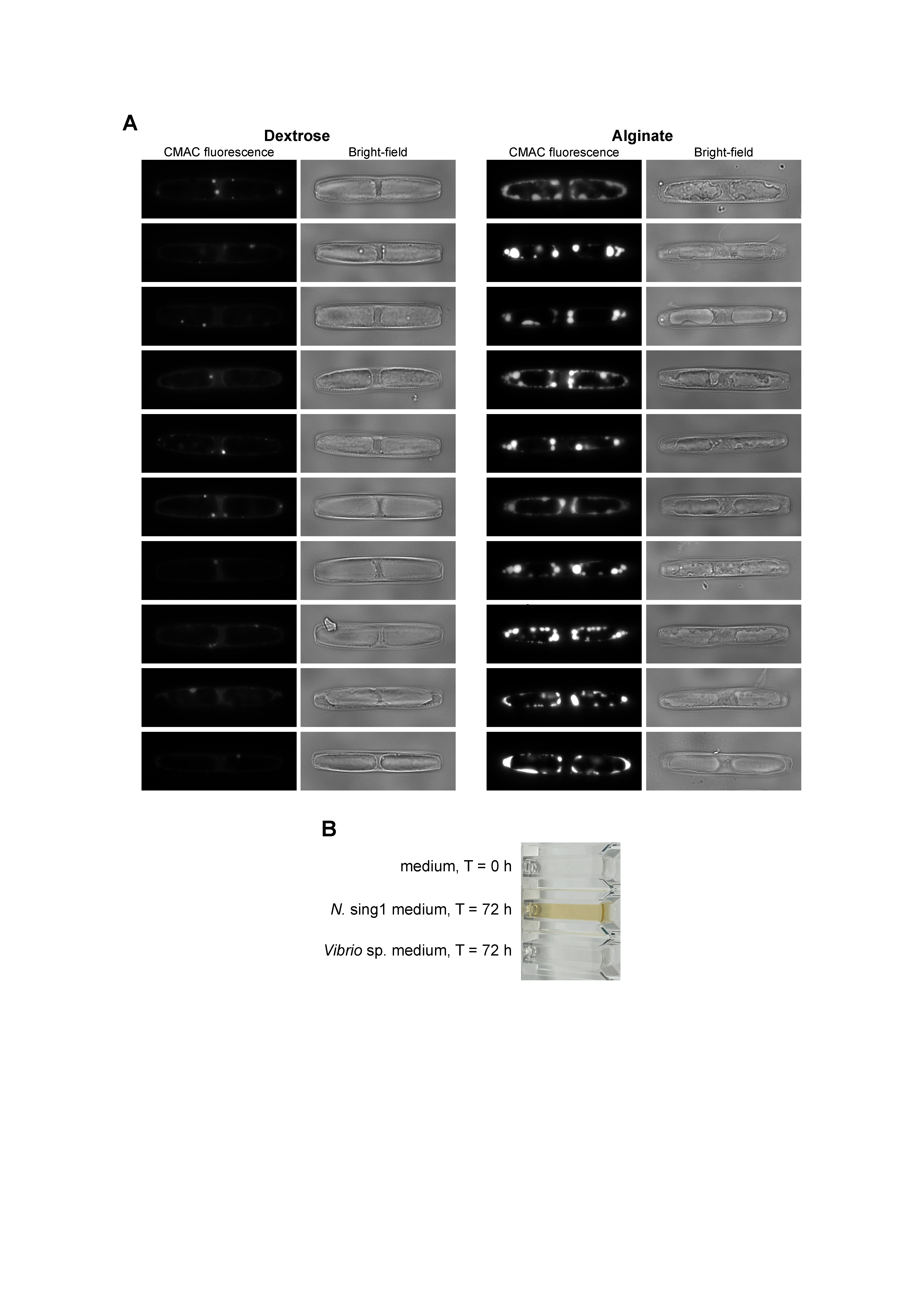

Supplement: S16 Fig — (A) Vacuole staining in diatoms grown on dextrose and alginate. CMAC dye shows an accumulation of vacuoles in diatoms grown on alginate, but not on dextrose (n = 10). (B) Culture medium undergoes yellowing over time in N. sing1 cultures (middle, 72 h), but not in Vibrio sp. culture (bottom, 72 h). (TIF) [file pbio.3003038.s016.tif]

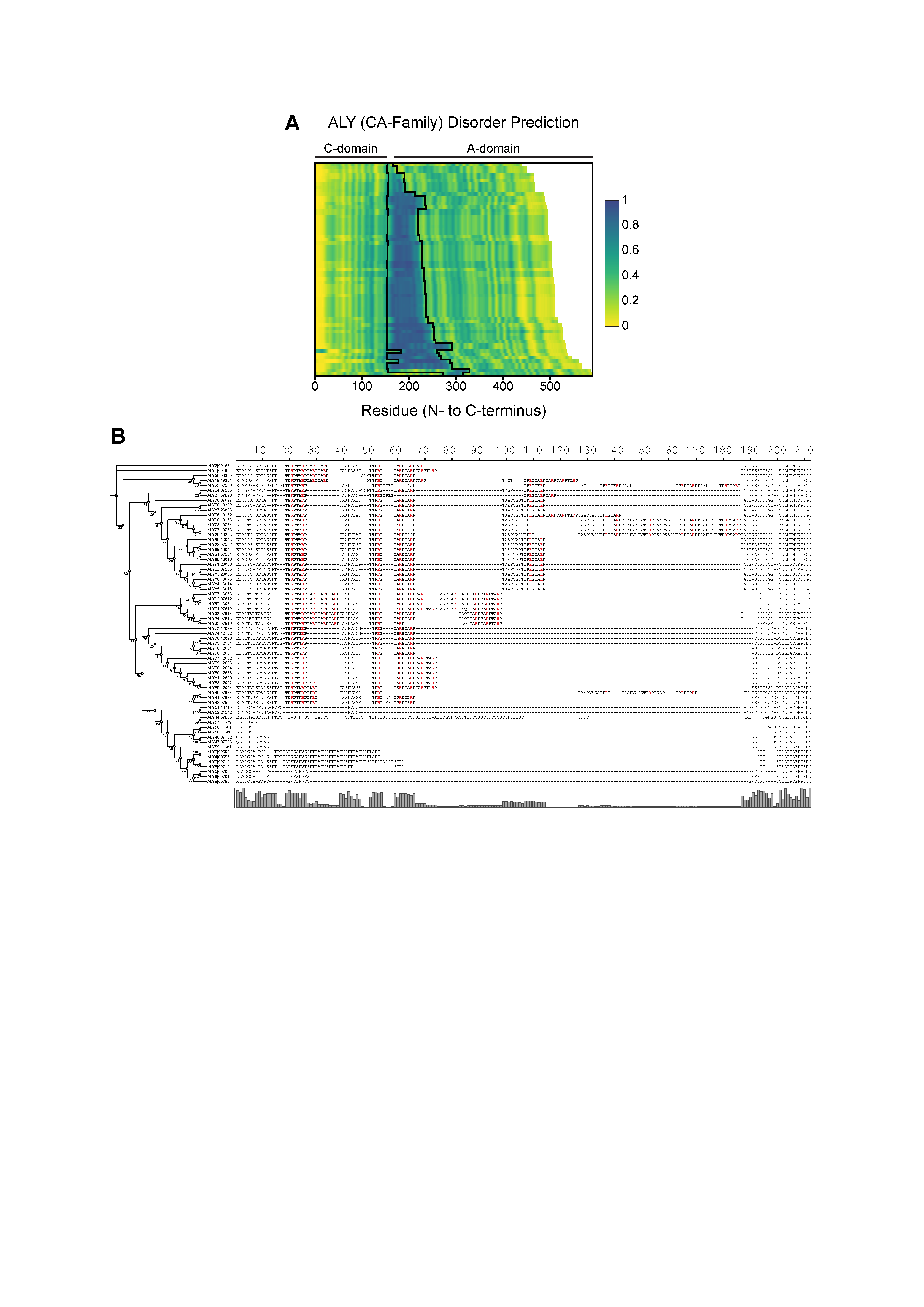

Supplement: S17 Fig — (A) The heatmap shows the predicted disorder scores (IUPred3) for N. sing1 CA family ALY proteins (green/blue scale). Note that the disordered regions are variable in length and occur between the C- and A-domains. The black lines denote the boundary formed by the annotated C- and A-domains. The data underlying this figure can be found in S10 Data. (B) Maximum likelihood phylogenetic tree (bootstrap replicates = 1000) and protein sequence alignment of N. sing1 CA family ALY TARP repeat region. The alignment was manually generated to highlight how TARP repeats vary in tetrapeptide unit lengths between closely related ALYs. TARP repeats are in bold and arginine residues are highlighted in red. The data underlying this figure can be found in S10 Data. (TIF) [file pbio.3003038.s017.tif]

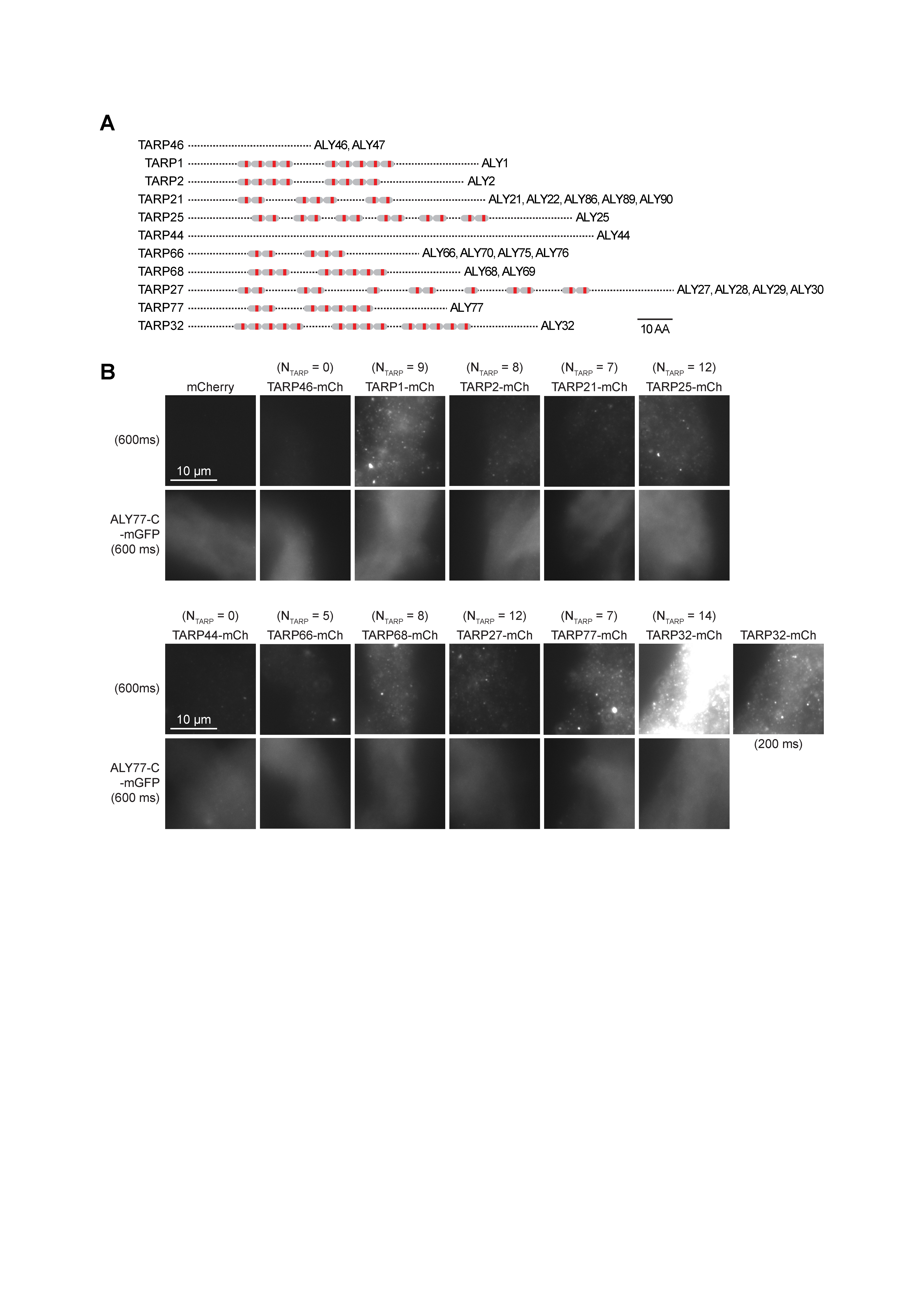

Supplement: S18 Fig — (A) The cartoon illustrates the set of TARP repeat regions synthesized as gene constructs. These constructs are named after the first ALY gene they are found in (e.g. TARP1 is found in ALY1). ALY genes with the TARP repeat regions are listed on the right. TARP repeat units are shaded in gray while the red line represents the arginine residue. (B) Double staining of alginate hydrogels with recombinant TARP-mCherry and ALY77-C-mGFP fusion proteins. NTARP denotes the number of TARP repeats present in the recombinant protein. Brighter signals are observed in recombinant proteins that contain more TARP repeats. Samples were imaged at 100× magnification (600 ms exposure) using the TRIT-C (mCherry; top row) and GFP (mGFP; bottom row) channels (0 low–800 high). For TARP32-mCh, imaging was performed using a shorter exposure (200 ms) due to signal saturation. Scale = 10 µm. (TIF) [file pbio.3003038.s018.tif]

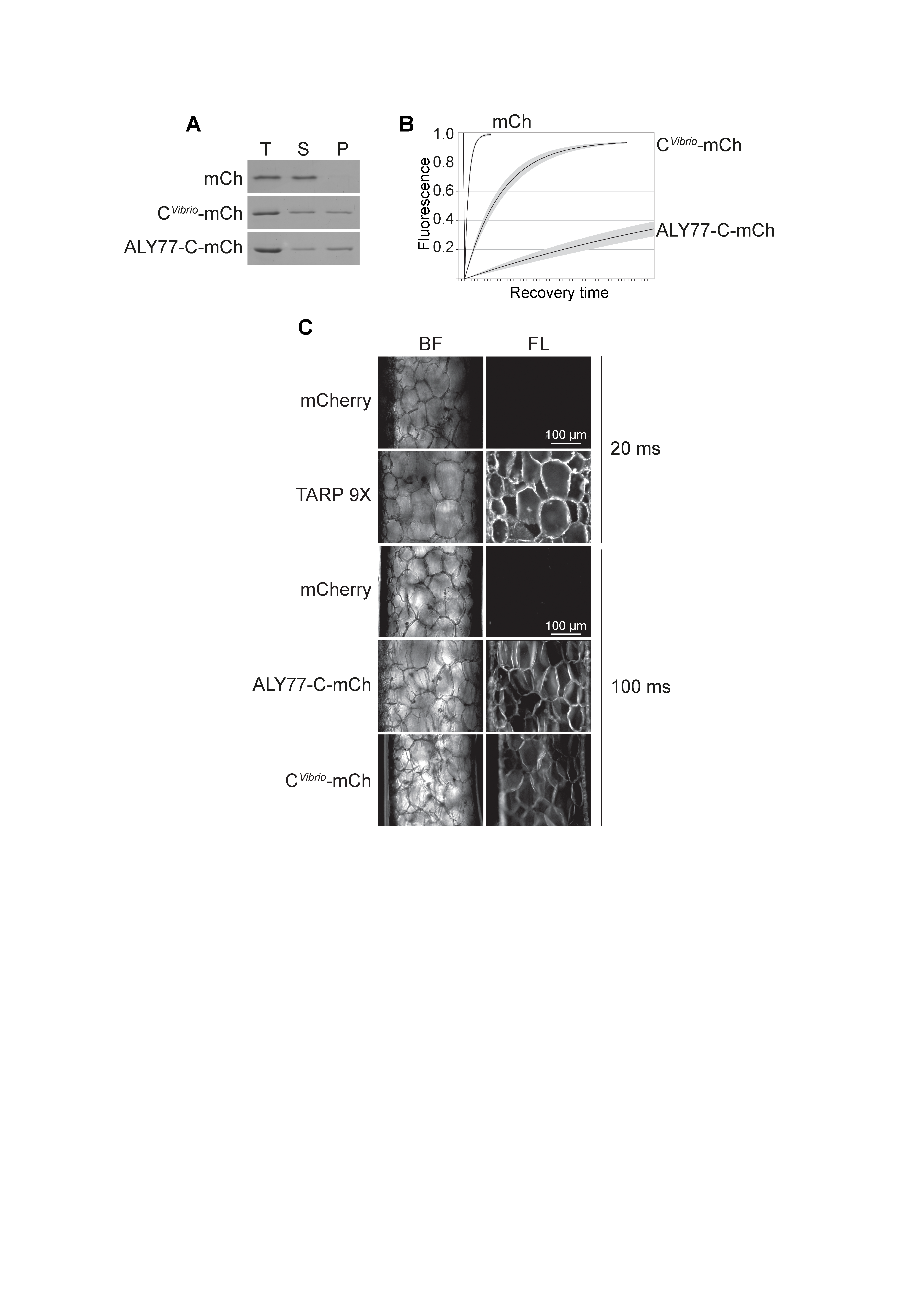

Supplement: S19 Fig — (A) SDS-PAGE of total (T), supernatant (soluble) (S) and pellet (insoluble) (P) fractions from an alginate pelleting assay using ALY77-C-mCh and CVibrio-mCh recombinant proteins. A protein band is observed in the pellet fraction for both C-domain fusion proteins but not mCherry, indicating that both C-domains bind to alginate. (B) Fluorescence Recovery After Photobleaching (FRAP). The recovery time for both C-domain fusion proteins following bleaching is slower than mCherry, indicating that both C-domains associate with the alginate gel. A stronger association for ALY77-C-mCh compared to CVibrio-mCh is observed. The data underlying this figure can be found in S12 Data. (C) Sargassum tissue binding assay using C-domain fusion proteins and TARP 9X-mCh recombinant protein. All three fusion proteins bound to the cell wall of Sargassum tissues, as seen from the signal when tissues were visualized under epifluorescence. No signal was observed in the mCherry control. Scale = 100 µm. (TIF) [file pbio.3003038.s019.tif]
