# Supplementary material for: Diatom heterotrophy on brown algal polysaccharides emerged through horizontal gene transfer, gene duplication, and neofunctionalization
Source: PLoS Biol. 2025 Apr 1;23(4):e3003038. doi: 10.1371/journal.pbio.3003038 (PMC11960938; doi:10.1371/journal.pbio.3003038)
Supplement: Attachment — Submitted filename: Lim et al. Response-to-Reviewers.pdf [file pbio.3003038.s041.pdf]

RESPONSE TO REVIEWERS

We thank the reviewers for their comments and helpful suggestions for improvement of our manuscript. Please find our responses below.

Summary of Major Changes

1. Additional information added to the revised manuscript: The original manuscript showed data suggesting that the 15 bp insertional mutation in the A^nC^n Alginate lyases converted them from an endolytic to exolytic mode of action. This is supported by structural modeling (Fig. 4C) and enzyme activity assays showing that the insertion-containing enzyme is indeed exolytic (Fig. 4D and E). We have now analyzed a mutant version of this enzyme where the insertion has been neatly removed. We find that this enzyme is shifted to a predominantly endolytic mode of action. This adds definitive evidence concerning the effect of the insertional mutation and the new data have been included as Fig. 4F.

2. Based on recurring reviewer comments, we have revised the presentation of Figures 2 and 3 to clarify the presentation and provide additional information that should improve readability.

Point-By-Point Responses

Reviewer #1

Review of 'Innovations in Alginate Catabolism Leading to Heterotrophy and Adaptive Evolution of Diatoms'

This excellent paper sequences the genome of a heterotrophic diatom that has recently lost photosynthetic function to become a heterotroph feeding off alginate provided by seaweeds. The aim of the work is to understand how this change in lifestyle was underpinned by genomic and functional adaptations.

The authors produce a high-quality genome assembly of the diatom. Analysis of the genome identifies a highly duplicated gene family predicted to function in the breakdown of alginate. They then show this gene family was gained by HGT from marine bacteria and then subject to numerous rounds of gene duplication. They nicely demonstrate that this pattern of gene duplication is the product of gene transposition.

They then depart into a series of experiments to investigate the function of these paralogue families. The authors confirm the function of the gene (alginate breakdown) but also demonstrate that multiple paralogue families have diversified into differing functions including binding and transportation of alginate.

The quality in this paper is very high. They conduct a series of experiments to investigate a number of related questions that often arise from analysis of HGT events, for example they test the possibility that the [approximate] bacterial donor gene secretion signal peptide would function in a eukaryotic system. It does, and so they

add considerable weight to the idea that the HGT they have observed would lead to a direct gain of function.

I think this is a really good piece of work, few explorations of HGT include this attention to functional biology of the HGT family, I only have minor suggestions.

1. I think the phylogenies showing the HGT from bacteria should be a primary figure not a supplementary figure.

We agree and have expanded the tree documenting HGT in Fig. 1C. There is now a substantial increase in the number of species shown in the primary figure. Because of this we have opted to remove an additional tree from the supplementary data.

2. Paragraph 298-315. When discussing co-localisation with lysosome. I don't doubt this data, but firstly here I think the authors should state in this section that this is assayed by co-localisation with Lyostracker (unless I missed something). This information is in the methods but should be stated here as well. I think the authors should also add a caveat that Lysotracker in diatoms can also stain a range of acidic vesicles, some associated with exoskeleton formation, so it is not 100% reliable for pure lyososomes organelles as understood for animal cella. Only a brief caveat is needed.

We apologize for the confusion. While we used Lysotracker in the experiments with animal cells, the experiments with diatoms were conducted using CMAC (7-amino-4-chloromethylcoumarin). We have revised the text to identify the dye being employed in each case.

3. Line 315 'which is consistent with the behaviour of a eukaryotic import receptor'. Please add a reference.

We agree that this statement calls for a reference. However, upon review we feel that it is overly broad and have amended the sentence to: “Overall, this trafficking pattern is consistent with the proposed alginate import function.” (Lines 424-425).

4. Very minor point - line 582-586. I had to reread this section several times to get a sense of how they processed the alignment sequence gather for their phylogenetic tree analysis. My suggestion is that the authors just reread and may be edit this section for clarity.

We agree and have revised the section to add additional information on how the trees were constructed.

<u>Reviewer #2</u>

The manuscript by Lim et al. describes the discovery and analysis of alginate catabolism in a diatom (Nitzschia sing1) that has lost the ability to carry out photosynthesis. The paper is well-written, combines multiple innovative techniques and analyses to document an initial horizontal gene transfer that was followed by multiple gene duplication events and enzyme neofunctionalization. I congratulate the

authors for their extensive and well-described study. I have only minor suggestions.

1. Title - as written, the title implies that alginate catabolism is what led to the loss of photosynthesis by these diatoms. This possibility was not really the topic of the manuscript. I suggest that the authors modify the title to more accurately describe the findings of their paper.

We agree and have revised the paper's title to more accurately reflect the main findings of our study: "Diatom Heterotrophy on Brown Algal Polysaccharides Through Horizontal Gene Transfer, Gene Duplication and Neofunctionalization".

2. Line 465. The authors state "Diatoms are likely to possess features that predispose them to a successful transition to obligate heterotrophy." If this was the case, then more diatom lineages would be expected to be obligate heterotrophs, which is not the case. I suggest the authors tone down this statement.

We agree and have revised the statement to: "Diatoms possess features that are likely to have supported the transition to obligate heterotrophy." (Lines 586-587).

3. Fig. 2A legend. I could not find the "shaded in gray" in figure that the authors indicate in the legend.

We thank the reviewer for pointing out this mistake. We have revised Fig. 2A to properly highlight the monophyletic clade containing the $A^{n\text{-}TMD}$ and A^nC^n families.

Reviewer #3

The study "Innovations in Alginate Catabolism Leading to Heterotrophy and Adaptive Evolution of Diatoms" by Lim et al. is a beautiful piece of work that reveals a fascinating biological story about how evolution is connected to the ecology and biochemistry of diatoms. It was a pleasure to read and will be relevant and exciting for others, too. Through genome sequencing and phylogenetic analyses, the authors show that the diatom Nitzschia sing1 acquired alginate metabolism through an ancient horizontal gene transfer (HGT) event from bacteria. This sequence then diversified within the diatom through gene duplication, resulting in 91 different homologs. This phenomenon is frequently observed in bacterial populations degrading chitin (Datta, 2016) or alginate (Hehemann, 2016), but the mechanism of evolution is HGT in bacteria, and the exact role of these different enzyme homologs remains largely unclear. By providing various types of enzyme activity assays (NMR, binding, imaging), the authors demonstrate functional differences between the diversified sequences and present compelling evidence that these sequences diversified to adapt to novel functions, such as exo-active mode and binding. For example, they identify a 15 bp insertion in some sequences that could convert endo- to exo-enzymes or, along with other mutations, enhance alginate binding. Overall, the study addresses a very important question about the evolution of carbohydrate metabolism, and all major conclusions by the authors are well-supported by high-quality data and experiments. I recommend publication with only one major edit concerning writing and data representation, which I am confident the authors can address.

1. My main point is that I am essentially lost between Figures 2 and 3 (lines 182-217) due to the overall data visualization, narrative in the text, and unclear scientific logic. I believe this section needs reworking in both the text and figures. Here are some specific points:

We agree and have substantially revised the presentation of Figs. 2 and 3 to clarify and add more information.

2. In Figures 1 and 2, the trees lack branch lengths, and Figure 2 does not have leaf labels, which makes it difficult to follow the authors' reasoning without referring to the tree in Subfigure 4. It would be beneficial to make the trees more reader-friendly by adding metadata, such as domain organization or activity data, directly onto the tree as a heatmap or similar.

Our goal with Fig. 2 is to provide an overall understanding of the tree and how it groups the three ALY families (comparing panels A and B). For this purpose, we have employed a cladogram as opposed to showing branch lengths as this allows a better connection between panels A and B. In this case, we show bootstrap support as it provides a fundamental insight into the origin of the two subfamilies. We have added additional text to explain the rationale behind constructing the tree (related to point 1). The large number of leaves in the tree precludes direct labelling which remains accessible in the supplementary tree. We have added extra graphics to show the connection between panels A and B. We have also added additional graphics to the supplement to show the ALY gene structure and domain organization next to each leaf.

3. Figure 3 is particularly hard to understand. I don't see the need for Panel A, which merely shows gene orientation; there are few inversions, so what am I supposed to take away from this? Panel B is intended to explain the logic of unequal crossing over, but I needed to read up on this to comprehend the illustration, as it is not explained in the main text.

We apologize for the lack of clarity and have endeavored to provide additional information to both the figure and text. The figure is important as it supports some of the main conclusions regarding gene duplication through unequal crossing over and transposition. To provide for a better flow of information, we have added a new panel A that shows the distribution of the ALY loci on contigs. Panel B is important as it shows that the majority of loci contain genes in an uninterrupted head-to-tail orientation, which provides evidence for gene duplication through unequal crossing over. This is further supported by intergenic homology which is shown in panel C and S5 Fig. With regards to the logic of unequal crossing over, we have expanded the cartoon and added additional information in the text to make the overall presentation more accessible. The grouping of loci sharing genes related by 100 percent bootstrap support (Fig. 3B) is used to support duplication through transposition (note that we have corrected two errors detected in the figure from the original submission). The text regarding this section has also been expanded to provide better explanation.

4. The text section lacks a clear statement that these sequences evolve through duplication. I expect higher intra-sequence homology of A domains in AnCn

sequences than between different sequences. While this is visible in Supplementary Figure 4, it is not clearly established in the main text or figures. The authors should clarify this point. There are other systems where additional protein domains evolve through recombination or HGT with other regions.

Fig. 2B shows all the duplicated domains graphically. Because all of the A^nC^n A-sequences are grouped in the Fig. 2A tree it follows that there is higher intra-sequence homology. This can be seen both in the primary Fig. 2A where 2B gives the domain organization, or in S3 Fig. where each leaf is labelled.

The role of domain duplication is highlighted in the following passages:

Abstract (lines 27-30)
"By contrast, the two others underwent **domain duplication**, gain, loss, rearrangement, and mutation to encode novel functions…"

Results (lines 191-194)
"Based on these findings, we can further infer other mutational events leading to the two derived families—these include domain loss ($A^{n\text{-}TMD}$ family), **duplication** ($A^{n\text{-}TMD}$ and A^nC^n families) and acquisition (transmembrane domains of $A^{n\text{-}TMD}$ family)."

Discussion (lines 491-495)
"From this basis, the capacity to degrade alginate evolved through gene duplication and neofunctionalization. Unequal crossing over and transposition underlie duplication (Fig. 3), while **domain duplication**, gain, loss, mutation, and de novo sequence evolution led to the advent of new protein activities."

Discussion (lines 525-527)
"Unequal crossing over can account for gene duplication as well as the **domain duplications** required for the emergence of $A^{n\text{-}TMD}$ and A^nC^n families (Fig. 2 and 3)."

5. From Supplementary Figure 4, it appears that the endo-lyases are less diverse, while there is more rapid evolution/diversification in Atmd and AnCn proteins, which aligns with the overall hypothesis of adaptive radiation in these sequences. Why is this not mentioned in the main text?

The paper is broadly divided into two sections. In the first, we establish ALY gene acquisition through HGT (Fig. 1), subfamily structure and phylogeny (Fig. 2) and duplication through unequal crossing over and transposition (Fig. 3). In the second we focus on obtaining experimental evidence that demonstrates the link between sequence variation and new functions (Figs. 4-6). While we agree that branch length variation and general sequence divergence are likely to be supportive of our overall conclusions, we choose not to emphasize this approach since it does not provide conclusive evidence regarding the genetic basis of new function.

6. In line 186, they state "orientation that conforms to duplication through unequal crossing over," but in line 201, they discuss transposition. These are different mechanisms of gene duplication, and it is unclear how the authors distinguish between the two scenarios given the data they have.

We apologize for this confusion and have endeavored to clarify these points. The presentation has been expanded to better explain how we distinguish the mechanisms of gene duplication. Briefly: Tandem duplication through unequal crossing over is supported by Fig. 3B and in particular 3C, which provides definitive evidence. Duplication through transposition is defined as follows: "…we examined the phylogenetic relationships between genes encoded at the various ALY loci (Fig. 3B) and assigned a possible relationship through transposition when genes at two or more unlinked loci (separated by unique sequences greater than 10 kb in length) form monophyletic clades with substantial statistical support (100% bootstrap support). Employing a stringent cutoff identifies such events with confidence but is also likely to exclude transposition-derived loci where greater sequence divergence has occurred between genes." (Lines 256-262).

7. It is difficult to see in Supplementary Figure 4, but it seems that there is also a small amount of sequence divergence in different A domains within one AnCn protein. Why is this not discussed in more detail? Would it not make sense to analyze the rate of evolution within the A domains before examining the inter-gene regions? Additionally, a clear comparison of the evolution of the A domain versus the IG domain is lacking. What type of information can be learned from the similarity of the A domain, and why is it necessary to look at the IG domain? As I am not a geneticist, more explanations would help me follow the argument.

Our response is related to point 5. We believe that the data presented fully support the paper's main conclusions, which would not be substantially strengthened by analyzing raw sequence variation (without assigning function to that variation).

With regards to the choice to focus on intergenic homology: These findings provide definitive evidence for duplication through unequal crossing over. They further permit us to conclude that the evolution of alginate catabolism is ongoing. We find this to be interesting as it generally supports the notion that there is significant functional diversity evolving at the genus and species level. We elaborate on this in the discussion: "Tandem duplicates commonly constitute a significant proportion (~10 %) of eukaryotic gene content [88–90] and their expansion has been associated with the evolution of environmental sensing in plants [91] as well as adaptation to benthic marine habitats in diatoms [40] and shrimp [92]. To the best of our knowledge, intergenic homology has not been employed to identify recent duplication events as shown here for ALY loci (Fig. 3C and S5 Fig). The systematic application of this approach has the potential to identify actively duplicating loci related to ongoing adaptive change." (Lines 532-539).

8. Line 21: The phrase "manner of energy acquisition is unclear" is confusing; it states in the same sentence that it is a heterotroph. Please specify what is unclear.

We agree and have re-phrased this passage: "However, in the genus *Nitzschia*, loss of photosynthesis led to a group of free-living secondary heterotrophs whose manner of acquiring chemical energy is unclear." (Lines 20-21).

9. Line 111: Where was growth on seaweed polysaccharides shown?

These citations were given in the introduction, for clarity they have also been added to this passage: “Because apochlorotic diatoms grow on diverse seaweed-derived polysaccharides [30,31,33,34], we searched the *N.* sing1 genome for carbohydrate-active enzymes (CAZymes) [42].” (Lines 111-113).

10. Lines 431-433: Are the monosaccharide catabolic genes known to metabolize acidic sugars in bacteria absent in this diatom?

The statement is correct, we find no evidence for homologs of bacterial exolytic alginate lyases (OALs). Please see lines 284-286: “Similarity searches failed to return any clear *N.* sing1 homologs of bacterial transporters or OAL enzymes (S5 Table), suggesting that *N.* sing1 executes these steps through distinct machineries.”

11. Line 456: Why was Sargassum chosen? In line 128, you mention it as an epiphyte of brown algae; which one? I had to refer back to reference 32 for clarification, but it would be better to include these details in the main text.

We apologize for the confusion. We have added the following referenced statement to the results section to clarify why *Sargassum* was chosen. Lines 156-157: “*N.* sing1 occurs as an epiphyte on brown seaweeds (*Fucus serratus* [34] and *Sargassum sp*. [33]) and can catabolize alginate [33].”

12. Figure 2: I don't see the gray shaded area intended to highlight Antmd or AnCn domains.

We thank the reviewer for pointing out this mistake. We have properly highlighted the monophyletic clade containing both $A^{n\text{-}TMD}$ and $A^{n}C^{n}$ families.

RESPONSE TO EDITOR

1. Please make your Title more explicit and appealing to a broader readership. We suggest "Gene transfer from bacteria enabled Nitzchia diatoms to switch from photosynthesis to heterotrophic consumption of brown algal polysaccharides"

We have revised the title to **"Diatom Heterotrophy on Brown Algal Polysaccharides Through Horizontal Gene Transfer, Gene Duplication and Neofunctionalization"** to more accurately reflect the main findings of our study.

2. Please address my Data Policy requests below; specifically, we need you to supply the numerical values underlying Figs 1A, 2A (treefile), 3B, 4AB, 5BDE, 6C, S1, S2, S3 (treefile), S4 (treefiles), S6, S7BC, S10, S12A, S14B, either as a supplementary data file or as a permanent DOI'd deposition.

The numerical values underlying Figures 1A, 2A (tree file), 3B, 4AB, 5BDE, 6C, S1, S2, S3 (tree file), S4 (tree file), S6, S9BC, S10, S12A and S14B have been provided as supplementary data files (SX Data).

- S1 Fig. raw data can be found in S1 Data
- Fig. 1A, S2 Fig. raw data can be found in S2 Data
- Fig. 1B raw data can be found in S3 Data (previously labelled as S4 Table)
- Fig. 1C raw data (.tree file, Newick format) can be found in S4 Data (zip compressed file)
- Fig. 2A, S3 Fig. (previously S4 Fig) raw data (.tree file, Newick format) can be found in S5 Data (zip compressed file)
- Fig. 3C (previously Fig. 3B), S5 Fig (previously S6 Fig). raw data can be found in S6 Data
- Fig. 4A, 4B raw data can be found in S7 Data
- S7B, S7C Fig. (previously S9B, S9C) raw data can be found in S8 Data
- Fig. 5B, 5D, 5E raw data can be found in S9 Data
- S8 Fig (previously S10 Fig). raw data can be found in https://zenodo.org/records/14410447
- S10A Fig. (previously S12A Fig.) raw data can be found in S10 Data
- Fig. 6C raw data can be found in S11 Data
- S12B Fig. (previously S14B Fig.) raw data can be found in S12 Data

3. Please cite the location of the data clearly in all relevant main and supplementary Figure legends, e.g. "The data underlying this Figure can be found in S1 Data" or "The data underlying this Figure can be found in https://zenodo.org/records/XXXXXXXX

We have cited the location of the data clearly in all relevant main and supplementary figure legends.

4. Please make any custom code available, either as a supplementary file or as part of your data deposition.

Custom code is deposited in (https://zenodo.org/records/14601569).
